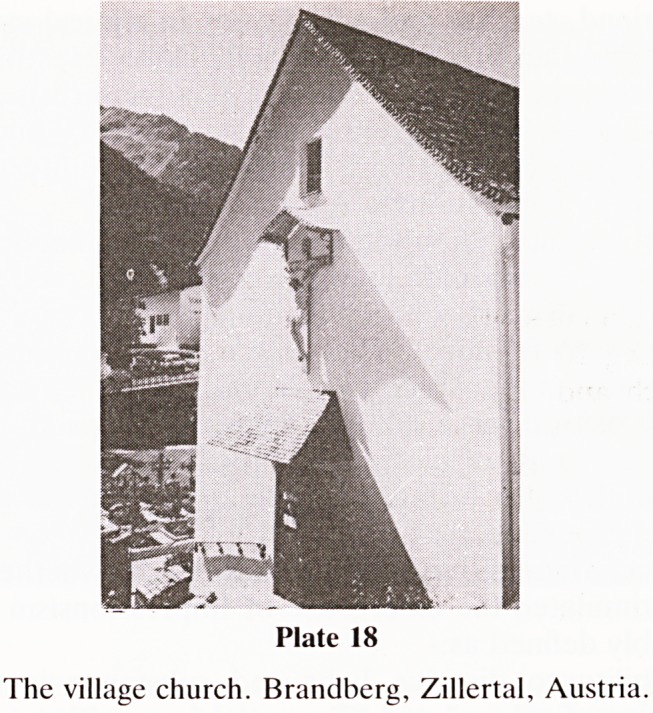# Impressions

**Published:** 1989-11

**Authors:** A John Webb

**Affiliations:** Presidential Address of the Medico-Chirurgical Society delivered on 12.10.88


Bristol Medico-Chirurgical Journal Volume 104 (iv) November 1989

### IMPRESSIONS

**A John Webb, ChM, FRCS, FIAC**
Presidential Address of the Medico-Chirurgical Society delivered on 12.10.88

Members of the Society and guests.
In 1951 when Mr Jackman was President of this Society, I as one of his dressers, was permitted to attend my first 'Med-Chi' meeting. I was overawed. The 'Med-Chi' was, after all, the premier postgraduate society of the City and far beyond. I remember how fascinated I was to observe the proceedings and to register the elegant eminence of the members. Little did I consider then that I might emulate 'Jacko' and for this honour I am grateful to you all.

The title of my address is—'Impressions' and I hope to range through a variety of scenes, of people and events which have impressed me and enriched my experience. Naturally reminiscence and recollection is involved and I have worried at that. Boring and uninteresting anecdotal recall is awful: so may I state at the outset that it is my wish to interest, amuse and perhaps even affect you by what I have to say.

I have sought justification for reminiscence from some perceptive observers; many qualify, but I have chosen two. Thomas Stearns Eliot.

> Time present and Time past are both perhaps
> present in Time future and Time future
> contained in Time past.
> Four Quartets. Burnt Norton.

Secondly and more bemusing; Marcel Proust in his 'A la recherche du temps perdu'; his peon of delight from 'les petites madeleines'—those squat plump little cakes dipped into herb tea, so rekindled memories of his Aunt Léonie and his beloved village of Combray and enabled him to write with such artistry, brilliant psychological insight and painting of his characters. For Proust one needs time and concentration, but truly, it is worth it. (Proust. Remembrance of Things Past. Vol 1 p48. Kilmartin translation.) For him the past still existed.

To begin with, some impressions of my family and fore-bears to include many alive during my lifetime. They were mostly very ordinary people. We possessed our share of skeletons but none will be consciously revealed.

My maternal great grandfather Robert Moon 1846–1902, lived in Lilymead Avenue, Knowle (Plate 1). He owned and ran a tin printing factory near Temple Church and died of probably gastric cancer. His large funeral on 9th June 1902 was reported in the local press and because he was a Sunday School Superintendent at Wycliffe Congregational Church, some 300 scholars followed the cortege to Arnos Vale Cemetery and sang his favourite hymns at the graveside. On 20th May 1866 he had married Sarah Bruton at St Simon's Church Baptist Mills who survived him with five children, the penultimate was my grandfather Robert Henry Moon b.28.4 1875 in Colston Street. On 13.4.1898 he married Elizabeth Ashwin (of City Road) at Lodge Street Chapel (just behind the Colston Hall) who bore him five children, the penultimate being my mother Gwendoline, known to all as 'Queenie' (Plate 2).

Turning to the other side, my paternal Grandfather, Ernest John Francis Webb married Mary Harriet Coates at St Luke's Church, Bedminster on 17.8.1895 (Plate 3). My paternal great grandfather John Webb, had married Isabella Blackmore, a qualified teacher, at Wycliffe Chapel, Guinea Street on 23.12.1866.

The Coates family derived from Newcastle via Carlisle, where a branch of the family still resides. There were many

**Plate 1**

My great grandfather, Robert Moon 1846–1902.

**Plate 2**

My maternal grandparents, Robert Henry Moon and Elizabeth Ashwin. c. 1897.

**Plate 3**

Marriage of my paternal grandparents. Wedding photograph 1895. Oxford Street, Totterdown. My paternal great grand-father John Webb is standing hatless in the back row.

offspring from Thomas and Mary Coates, of whom Emily, Harriet, Richard and Will are the important 'white sheep' for my story. The mother Mary, had died young and Emily the eldest girl, not only brought up the others but trained as a teacher.

The wedding reception of my grandparents took place at the Coates home in Oxford Street Totterdown. They very soon moved, together with a sizeable population exodus from South Bristol to the newly built northern suburb of Bishopston. Their rented house was 47, Theresa Avenue and there, on 26.2.1897, my father, Charles Reginald was born after two previous still births. His mother had suffered damage from rheumatic fever and my father's survival was—according to Aunt Emily, who was present—fortuitous. Left to himself he happened to breathe! Not surprisingly he developed into a somewhat cossetted boy, adored by his sickly but in other ways sparkling mother. She was known to all as 'Polly'. She was vivacious, very pretty and possessed a lovely singing voice; which gift was passed on.

My father, after early private education, went to Sefton Park School where he made his mark as an actor. His prime role was the Mad Hatter in 'Alice in Wonderland'. He was very good at sport, fair at work, and well thrashed: keeping pigeons was his hobby. His formal education was completed at the newly opened Fairfield School where he was marched from Sefton in the first group of pupils (c 1908). Archie Leach (Carey Grant) attended the same establishment somewhat later.

Years after, in the 1960's, my father walked around Sefton Park School and encountered the names of his many friends recorded on the school memorial (1914–18). He remembered every one of them and their foibles.

Reg, as he was always known, was extremely fond of the Coates side of his family—they were the more artistic—and at a family wedding in 1907 when his favourite and most supportive Aunt Emily married an elderly widower from Gloucester—Charles Dancey—an important family gathering is recorded (Plate 4). The lads include my father and his two cousins, Fred and Ernie Coates—children of Will and Alice—both of whom subsequently achieved great success as men of business. Two uncles are present and both played significant roles in the story. Richard (Dick) a tall upstanding and delightful man, was the spiritual and financial supporter to his ailing sister Polly and my grandfather. He later married a Miss Boag from Carlisle and settled in Capetown S.A. He was blessed with one son, Alfred—and both were very successful businessmen. Sadly his wife died very young. The other was Will, a short, jovial, loveable, cigar smoking comedian, who lived successively on Ashley Down Road and Bromley Heath Road, Downend. I shall mention him again.

The family lived at 47 Theresa Avenue, Bishopston and for years were active at the newly formed Horfield Baptist Church. For my grandfather, Ernest, the Sunday School was special and he was Superintendent for many years. But in 1915—a fateful year—he was forced to resign because of Polly's failing heart. One of my treasured possessions is the illuminated book presented to him; signed by most of the scholars and teachers. Some of the scholars were listed 'in absentia' as being on active service—so many, never returned. On 15.7.1918, Polly died of heart failure, with Ernest and Emily in attendance. Her doctor was Norman Heron, father of Gordon, a former President of this Society. My father was just too late back from France.

We now move to my parents wedding group at 4, Downend Road, Horfield in the summer of 1928 (Plate 5). On 29.12.29 I arrived and on 3.1.1932 my eldest sister, Mary Elizabeth. I remember her as a lovely little girl, but it all came to an end on 15.1.1933 (Plate 6). She had been taken ill with alleged gastro-enteritis. The doctor admitted her to the Homeopathic Hospital and after a few days she died. I have, since then, fervently wished that she had gone to the Children's Hospital where she might at least have been given rectal tap water. Our house at the time was 30, Nevil Road and I recall very clearly the hordes of people who trooped down it to the County Ground.

It took many years to divine why I experienced a strong visual sensation—almost an aura—when reaching the crest of

**Plate 5**

My parents wedding group. Summer 1928; taken at 4 Downend Road, Horfield.

**Plate 4**

Marriage of Emily Coates to Charles Dancey 1907; Wedding group, 47 Theresa Avenue. My father and his cousins are the seated lads. Will Coates stands at the right, Dick Coates is seated near him.

**Plate 6**

My sister Mary Elizabeth and I, Autumn 1932. She is buried at Canford Cemetary.

**Plate 7**

The Moon Family, including my mother. Lizzie and Robert Henry (minus left arm) are seated.

**Plate 8**

Family group. The Firs, Easton-in-Gordano: Ethel cuddles my sister Julie. A lovely day in summer 1939.

Falcondale Road. It amounted to a powerful 'déja-vu' phenomenon. The explanation became obvious. Twice a week, for two years after Mary's death, my poor mother drove me in a push chair through Bishopston, Henleaze, Westbury on Trym to the grave at Canford. It was her physical and emotional battle to overcome our terrible loss.

Alleviation did arrive, for in 1935 Julie was born and in 1937 we were briefly reunited with the remarkable Aunt Emily Dancey who came over from Capetown for a holiday.

I now return to the wedding of 1928 to highlight the Moon family (Plate 7). My grandpa, Robert Henry, lost his left arm in an accident at the family factory because the normal press operator had gone to the war (1914). The limb was amputated through mid arm at the General Hospital and overnight and for decades to come that event constituted an economic near disaster. My grandmother—Lizzie—to whom I was very attached, developed progressive dysphagia in 1947 and months later was taken into the Homeopathic Hospital. Although assessed by R V Cooke she died a few days later. In due course as a medical student, I listened to Ronald Belsey harangue medical neglect in many cases of oesophageal cancer where the patient is late referred when the doctor's medicine cannot be swallowed. It so happened to Lizzie. Many years later when on the staff of the same hospital, I sought the notes for both Mary and Lizzie. Both had been torn out of the nicely bound record book! I was not surprised.

There were happy years in the late 30's and we lived in a cosy house, 1 Dongola Road. I resided mostly in the upper branches of my favourite apple tree and started at Sefton Park School. My father often took me to the County Ground and before the age of 10, I had seen Wally Hammond score several perfect centuries. My grandfather Ernest, had in 1929, remarried. Ethel Humfrys Maddox was the daughter of a former Bristol Corn Merchant and a Sunday School teacher at Horfield Baptist. She was an interesting and impressive lady. To me she was my paternal grandmother. I knew no other. Over many years she was very good to me. The couple moved to The Firs, Breaches Road, Easton-in-Gordano—which became for me a haven of delight (Plate 8). Ernest died there in 1955, aged 88—Ethel survived him by nearly twenty years. Ernest had worked as a Cartographer in Ordnance Survey, but since 1929, his life was that of contented, affluent retirement. He was a kindly, gentle man of strong non-conformist religious convictions. Reported to have been an impressive preacher, his handwriting was truly admirable and his standard grace at table I have adopted as my own.

An error of judgement coincident with September 1939 made us move from Dongola to 8 Berkeley Road, Bishopston. Other mistakes followed and the '39–45' war was punctuated by intermittent tragedies so that the halcyon days of the 1930's were sadly long gone. By September 1940, I was fortunate to enter Cotham School and I left in July 1948. Despite the 'Blitz' and other vicissitudes of the Second World War, my impression was of an excellent grammar school education which extended into academic sporting and cultural pursuits. It is a sadness to me that the state grammar school has gone. During the war I acquired two more sisters; in 1944 and 1946. At the time it was a shock, and to observe my father watching me play for the first XV accompanied by a baby in a pram required some personal and peer adjustment. They grew up into charming, thoughtful, talented and admirable girls and both have sustained and overcome great personal sorrows.

My grandfather Ernest was a moderate respiratory cripple (dating from the 1918 influenza epidemic) for the latter years of his life and died at The Firs on Easter Sunday 1.4.55. This house is now occupied by a delightful and considerate family who allowed me to look over the house in September 1988. It has been most tastefully modernised and bears a happy welcoming atmosphere.

My father Reg, was a supportive and inspiring father. In some ways his example was of the negative variety, and I regarded his life as one of unrealised and unfulfilled talent. His interest in literature and his gifts for oratory and singing were considerable but he was never allowed to enter his chosen career of the stage. Properly directed, he could have succeeded as a teacher of English and Drama. He possessed a fine ear for poetry, a perceptive interpretation of literature and left behind many notebooks of 'gems' abstracted from his reading. He was a very capable amateur operatic actor and his finest roles were Dr Engel in the 'Student Prince' and Dvorak in 'Summer Song' (Plate 9). Dating from the 'Trenches', too many cigarettes induced hypertension and chronic obstructive airways disease. In April 1971 he died in Budd Ward BRI from a massive stroke. It is appropriate at this time to state that my mother is an exceptional lady and her many qualities are beyond praise.

I have been blessed with a very happy marriage and four children, but the story of that is too long and exciting to tell. There are however two events of momentous importance. In 1961 when I was R.S.O. at the Queen Elizabeth Hospital, Birmingham, my eldest son, Mark then aged 4, became seriously ill with viral pericarditis. His life was nearly terminated by an unexpected sensitivity to Penicillin. Dr Otto Wolf had the good sense to 'stop all the treatment' and I shall never cease in my gratitude to him, for Mark survived. When

**Plate 9**

My father, 'Reg', as Dvorak in 'Summer Song', Bristol Light Opera Company c. 1954.

clinical progress is not being made, why not stop all the treatment (Plate 10).

By 1969, I had been a Consultant for 2 years and was content with my three children. I was assailed by a proposition; overcome by biology and in October 1969—a late child—a son, Jason, was born. He has been a great success and his presence has led me into interests which have transformed my life.

I now move to my next phase of impressions—1914–1918. Reg served in the Great War from 1915–1918 (Plate 11). My interest, which at times amounts to an obsession, was kindled by my boyhood 'chats', but largely his residual equipment: a tin hat, spurs, medals, puttees and above all two scruffy magazines; 'Fragments from France' by Capt. Bruce Bairnsfather. I knew all the cartoons. One classic example is "keep yer ead still or I'll have yer blasted ear off". Unsophisticated humour; clean, direct and so evocative (Plate 12).

Since 1960, my interest has surged and I have read many books as well as visiting the Somme battlefields. One poignant aspect of my story refers to Will Coates my father's uncle. When Reg enlisted he went to the Colston Hall. At his mother's pleading, Will went with him. At the door stood a Warrant Officer who was directing lads to different recruiting tables. Will placed two golden sovereigns in his hand and

**Plate 10**

My eldest son Mark with his godfather, now Professor Dennis Osmond. The Medical School, Summer 1964.

**Plate 11**

My father, right, with colleagues in the Royal Engineers; Cologne Army of Occupation 1919.

**Plate 12**

Cartoon; 'Fragments from France' by Captain Bruce Bairnsfather.

whispered—"put him in the Artillery his mother's got a bad heart". So, a perceptive uncle and a bribe enlisted Reg into the Royal Garrison Artillery. That day and thereafter most of his school and church friends were directed into the Infantry!—almost all were dead within a year. The memorial plaque on the Horfield Baptist Church Institute opened in 1921 portrays 39 names.

You have I expect heard the description of the British Soldiers as 'Lions led by Donkeys'. It is a quotation from Falkenhayn's memoirs—Hoffman speaking to his superior Field Marshall Lundendorff. The 'donkeys' were the British General Staff—dominated by cavalry officers; French, Haig etc. Now to just one episode in that terrible struggle. The Somme.

Nigel Jones in his admirable and quite unique book, 'The War Walk' (1983), describes the Great War as 'the savage rupture in the continuity of European Civilisation', and within the conflict the events which began at around 7 am on 1.7.16 in the district known as the Somme was the most devastating reverse the British and Empire armies have ever suffered (Plate 13). To walk now on the Somme front is both a beautiful and devastating experience. The countryside is magnificent in its richness and uncanny peace. How could so many hopefuls in Kitchener's army have fallen within 30 minutes of the start? The answer is plain for all to see. Open

**Plate 13**

An infantry trench, the Somme, 1st July 1916; preparing to go 'over the top'! Note the youth and modest size of soldiers in the foreground.

**Plate 14**

Stretcher Party, Autumn 1917, Passchendaele. Pilkem Ridge; the R.A.M.C. are certainly matching their motto—'In Arduis Fidelis'!

countryside and plenty of well dug in vantage points from which German machine gunner's could slay almost at leisure.

Two late veterans, known to me, are related to that sunny morning and to each other. G W (Bill) Hinton was the second master at Cotham School and for many years Chairman of Convocation, University of Bristol. I was privileged to treat him for malignancy many years before he died. Canon Percy Gay was a renowned Bristol prelate and Vicar of St George, Brandon Hill for many years. My only uncle, Bob Moon, was once a Church Warden there. Percy was a personality by any standards and was for a while Chaplain to Q.E.H. School. My eldest son, Mark, was a Bluecoat, so I have attended many school services conducted by Percy.

In 1916, both men were infantrymen with the Devons and I have it on sound authority that both went 'over the top' that morning; Bill was wounded in the leg and Percy carried him back from 'No Mans Land'. The finest Remembrance Day homily I have ever heard came from Percy. A casual, but deeply moving chat—especially for the boys. After all he had been there! That particular service was doubly rewarding. The motet performed by the school choir and orchestra was by a composer who until then was unknown to me: Claudio Monteverdi. The boys sang 'Beatus Vir' and it was and is a beautiful revelation.

The third battle of Ypres—'Wipers' to the soldiers—is generally regarded as exceeding the Somme in its horror. It is often known as just—Passchendaele—which is a village east of Ypres. John Masters (Fourteen-Eighteen) 1965 describes it as 'courage and sacrifice beyond understanding'. Such was the separation between the front line soldiers and their Generals that the whole event is rendered even more incomprehensible. In 1917, Sir Lancelot Kiggell who was Haig's Chief of Staff was driven forwards to the front. He is reported to have broken down into tears, "Good God" he muttered, "did we really send men to fight in that". One could echo this impression for the whole war (Plate 14).

In 1918, significant events affected my father, who was serving in the R.A. Communications as part of the Fifth or Reserve Army with its emblem of the Red Fox. This army came into effective being on 23.5.16 under the command of General Sir Hubert La Poer Gough. On 21.3.18 the full force of Ludendorff's Michael offensive set off from St Quentin putting 43 German divisions against 15 understrength British. Vastly out numbered, Gough fought one of the greatest retreats in history and was summarily sacked in April 1918 for so doing. My father's unit was destroyed but he had escaped through being at home on compassionate leave because of his mother's dire cardiac illness. He returned to a different unit. Gough was shabbily dealt with by Lloyd George and others and it was not until 1930 when Lord Birkenhead (F E Smith) published his book 'Turning Points in History' and devoted a whole chapter to Gough's 5th Army triumph against enormous odds that amends began. Gough died in March 1963 and his obituary in the Daily Telegraph was warm and complimentary to his personal qualities and dignity through years of adversity. His book, 'The Fifth Army' was bought by my father and is now my treasured possession. Incidentally the Fifth or 'Red Fox' Army is commemorated (1939!) without and within the entrance hall of St Mary's Hospital, Paddington.

Whenever I pass the bronze statue of Earl Haig on the close at Clifton College, I mentally cringe. I wish it was not there. The soldiers of 1914–18 deserved better from their Generals and my excuses for being interested in this period are that it was momentous and one cannot fail to be amazed at the enormous courage of the soldiers. The fact that it was an unbelievable waste of life and hope, cries out from the well kept and beautifully simple graveyards and memorials which remain.

The third element of impressions is the briefest and relates to my research. Brian Nicholas Brooke sometime Professor of Surgery at St George's Hospital, London and Reader in Surgery at the QEH, Birmingham in the early 1960's, is an artist of note; a man of wide culture, a surgical thinker of the highest quality and to crown it all, a sparkling personality. He invented the spout ileostomy as we know it today. He guided the surgical registrars in research and set me towards a project on studying lymph nodes draining colonic carcinoma. To further this, I returned to Bristol and consulted my friend and colleague Dennis Osmond; later to become Professor of Anatomy at McGill Univesity, Montreal. Dennis recommended that I tried lymph node impressions or imprints.

The impression technique on the freshly bisected excised lymph node involves touching the cut surface on to a dry slide in order to make a representative smear of the constituent cells. (Long Fox Lecture) 1973. The air dried smear is fixed in methanol and stained by May-Grunwald Giemsa or just Giemsa alone.

Imprints are one element of tissue cytology, and together with scrape-smears and fine needle aspiration biopsy have dominated and complemented my surgical pursuits without relaxation or respite since 1964. It is indispensable in surgical practice especially for breasts, goitres, salivary gland lesions, lymph nodes, soft tissue tumours and other sites. For me, it all began with Bryan Brooke and by a kind suggestion from Dennis Osmond.

My friend, teacher and adjudicator in clinical cytology is Paul Lopes Cardozo of Leiden and Delft. He thinks that devoted and competent Cytologists are either artists or artistically inclined—both pursuits are essential visual and involve spatial appreciations. Hence, with great humility, the final strand of my impressions is ART.

My artistic inclination was hazy until 1964. That Spring, my wife Audrie, wished to buy me a well deserved wedding present. In Moseley village, Birmingham, we saw a Monet print and both fell for it. The picture is, La Garenne Bezons so it dates from the Argenteuil period. Since then and almost as if that picture was a catalyst, I have developed a progressive interest in art of all times but unashamedly a particular delight in the French Impressionists, which are the subject of this brief excursion.

Art historians differ in their views as to whether earlier artists stimulated the emergence of Impressionsism which is reasonably defined as:-
"The ability to dissolve light and substance in dynamic abstraction of colour" and "To model form without recourse to definite outline". As far as I see it, J. M. W. Turner did all that and could well have been an important influence. I shall refer mainly to Oscar Claude Monet (1840–1926) as he is my favourite and his picture 'Soleil Levant' began it all.

Monet was a Norman by inclination who was brought to Le Havre from Paris at the age of 5 years. Nearby in Honfleur, lived Eugene Boudin (1844–1898) who tried patiently to influence the truculent, irreligious, headstrong, self indulgent but talented schoolboy, Monet. Boudin wrote, "everything painted directly and on the spot has a strength, vigour and vivacity of touch that cannot be attained in the studio".

Boudin painted many delicate sea and beach scenes around the Seine estuary. Monet succombed to Boudin's gentle persuasion and was also influenced by Victor Daubigny (1817–1878) especially for his river paintings, and a 'mad Dutchman'—Johaan Jongkind (1819–1891).

During the mid and late 1860's, Monet was extremely poor and the modest earnings from his output, which comprised largely sea and river scenes, went to pay his debts. From youth, Monet was a 'bon viveur' and this tendency lasted throughout his life. Some of the beach scenes, Beach at St Adresse (1867), Pointe de al Hève (1864) are especially admirable (Plate 15).

The Franco-Prussian War of 1870, induced Monet and his friend Camille Pissarro to avoid conscription by translating to England. Here both artists executed important paintings, several of which reside in the National Gallery (Westminster Bridge; Monet 1871; Lower Norwood, London. Pissarro 1870).

Both exiles returned to France in 1871. Monet went to live on the Seine at Argenteuil which was easily accessible from central Paris. Argenteuil, together with his wife and young family (1871–1878) was a productive and interesting period for Monet and so many subsequently famous pictures were inspired by the Seine, its boats and bridges, Bougival, La Grenopuilliére and its bathers. Like Daubigny before him, Monet often painted from a house boat, Le Botin, moored in mid stream.

In 1874, Impressionism and Impressionists were initiated by an art critic Louis Leroy in the magazine Le Charivari. An exhibition by Monet and his friends was the object of ridicule, especially Monet's painting, 'Impression Soleil Levant' (1872). Leroy coined the title 'Impressionistes' to mock these artists and the movement began.

Pierre August Revoir was one of the group. He showed an inclination rather more towards painting figures and scenes involving a particular 'locale' (Le Moulin de la Galette, 1876, La Loge, 1874). His beautiful female faces and sensuous dancers are so evocative. By 1879, Monet with his ailing wife Camille, accompanied by the recently widowed wife of a patron, Mme Hoschedé and her 5 children, moved further out to Vetheuil. It was a bad time for him as Camille died in 1879 and his pictures reflect his pathos in the winter scenes of dull sad light. His style altered a little; the brushwood became more dappled. His longstanding and supportive friend, Pissarro was then established at Pontoise and very productive. His style had also matured from the early influences of Corot and Courbet to the rich scenes around that small village (Les Toits Rouges, 1877).

By 1883, and soon after, Impressionists were becoming accepted and their pictures sought after, especially for Monet. He moved, with his mistress Agnes and entourage, to the village of Giverny. Initially he lived in a rented house but in 1886 moved to his eventually renowned residence, 'Le Maison du Pressoir'. By now he was famous. He painted, Poplars on the Epte (1891) and he painted a set scene at different seasons and in different lights. Some critics have criticised his abstract style at that time. It is a matter of taste.

His garden at Giverny was developed and water lilies were planted in the pond. The bridge was built and is immortalised on many canvases. The water lilies were the object of great attention and Monet was awarded a Government Commission to execute some enormous water lily canvases.

It was a huge undertaking, demanding from 1916–1926 to complete the pictures which fill the walls of two Salons in the Orangerie–Tuileries Museum. A cataract operation in 1923 nearly defeated him, but constant encouragement from his friend, 'Pere La Victoire', the 'Tiger of France', George Clemenceau, bore him up. What an achievement it was; for both of them. Soon afterwards Monet died—a hero—like his friend (Plate 16).

Ladies and gentleman you have all been very patient and attentive so I reach my final impression—the greatest impres-

**Plate 15**

Pointe de la Heve, Saint-Adresse 1864, Claude Monet.

**Plate 16**

An elderly Monet completing his 'Water Lily' series for the 'Orangerie', Paris.

**Plate 17**

Crucifixion; Cimabue (S. Domenico Arezzo c. 1820).

**Plate 18**

The village church. Brandberg, Zillertal, Austria.

sion of all. I am still striving to understand what it all means. Whether it is depicted on the wall of an Austrian village church in Brandberg Zillertal or by one of the greatest in early Italian artists, Cimabue, the message is the same

'Salvator Mundi'—to which I can only reply

'Salva Me'.

(Plates 17 and 18.)

**Footnote**

A significant component of this address was its visual content. A large number of both colour and black and white transparencies were projected. Some of the latter are shown here.

### REFERENCES AND SELECTED BIBLIOGRAPHY

PROUST Marcel 'Remembrance of Things Past' (Á la rechèrche du temps perdu). Translated by C. K. Scott Moncrieff and revised by Terence Kilmartin. Penguin Books—London 1983.
MASTERS John. 'Fourteen Eighteen' Michael Joseph—London 1965.
JONES Nigel. 'The War Walk' Robert Hall—London 1983.
GOUGH General Sir Hubert. 'The Fifth Army' Hodder and Stoughton—London 1931.
GRAVES Robert. 'Goodbye to all That'—Revised Edition. Penguin Books—London 1960.
WOLFF Leon. 'In Flanders Fields' Pan Books—London 1958.
CLARK Alan. 'The Donkeys' Mayflower Books—London 1964.
BIRKENHEAD The Earl of. 'Turning Points in History' (p230) Hutchinson—London 1930.
TAYLOR A. J. P. From Sarajevo to Potsdam' Thames and Hudson—London 1966.
TERRAINE John. 'The Great War 1914—1918' Hutchinson—London 1965.
WREN Jack. 'The Great Battles of World War I' Hamlyn—London 1971.
SEITZ William. 'Monet' Thames & Hudson—London 1984.
GAUNT William. 'The Impressionists' Thames & Hudson—London 1970.
PISCHEL Gina. 'The Golden History of Art' Golden Press—N.Y. 1968.
COURTHION Pierre. 'Impressionism' Translated John Shipley Harry N Abrams—N.Y. 1977.
SKEGGS Douglas. 'River of Light' Victor Gollancz—London 1987.
WELDER Diane. 'The Great Book of French Impressionism' Abbeville Press—N.Y. 1980.
BLUNDEN Marie & Godfrey. 'Impressionists and Impressionism' Rizzole—N.Y. 1976.
WEBB A. J. 'Through a Glass Darkly' Bristol Medico-Chirurgical Journal *89* 59–67. 1974.

## Figures and Tables

**Plate 1 f1:**
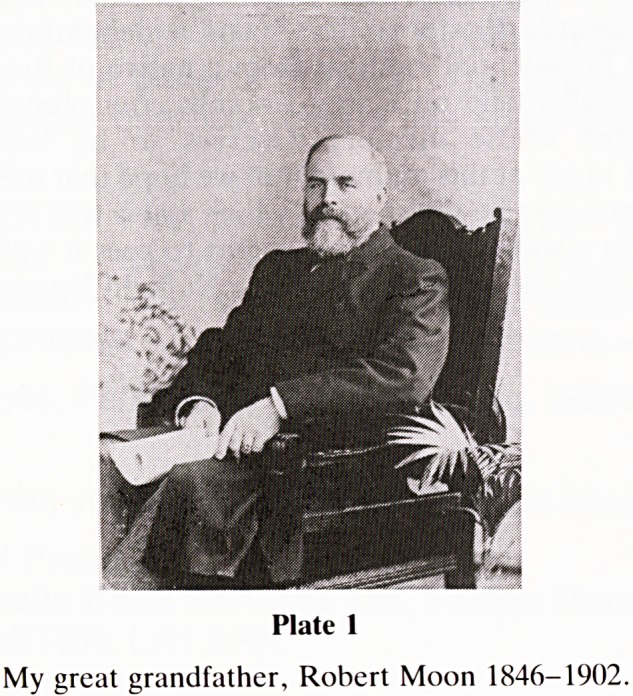


**Plate 2 f2:**
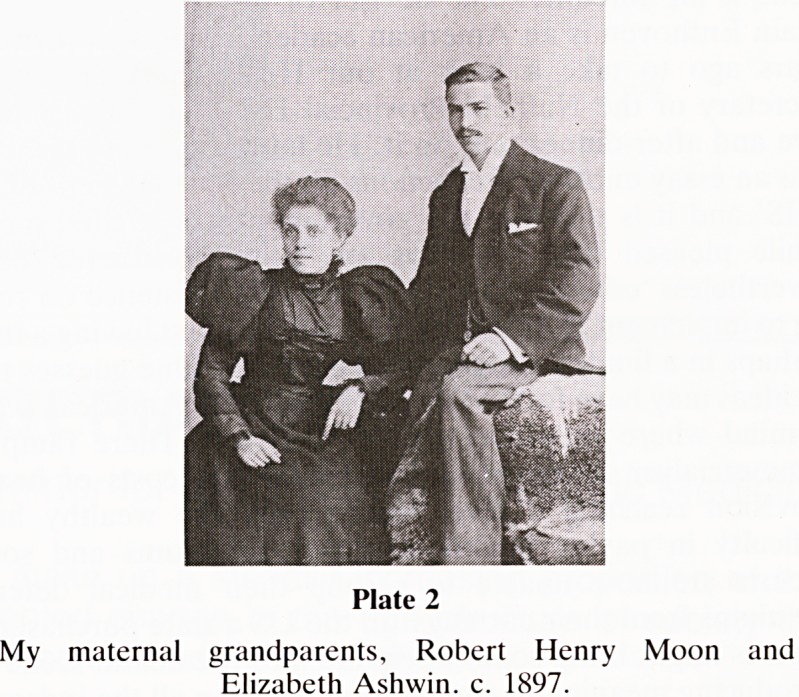


**Plate 3 f3:**
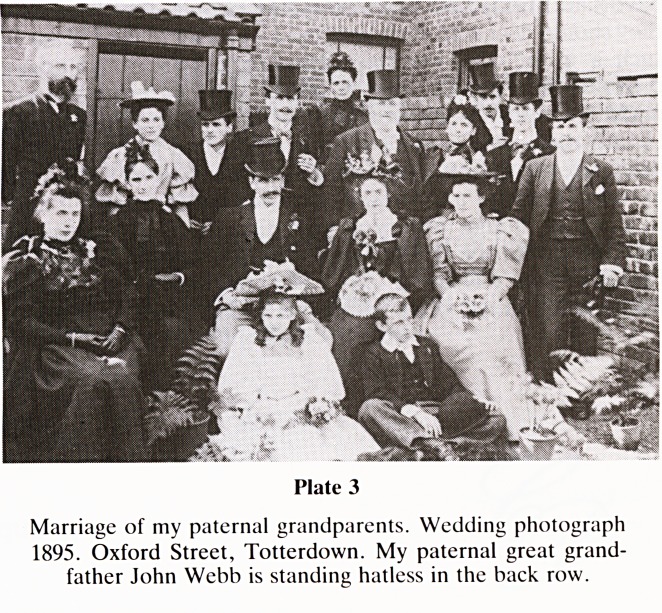


**Plate 4 f4:**
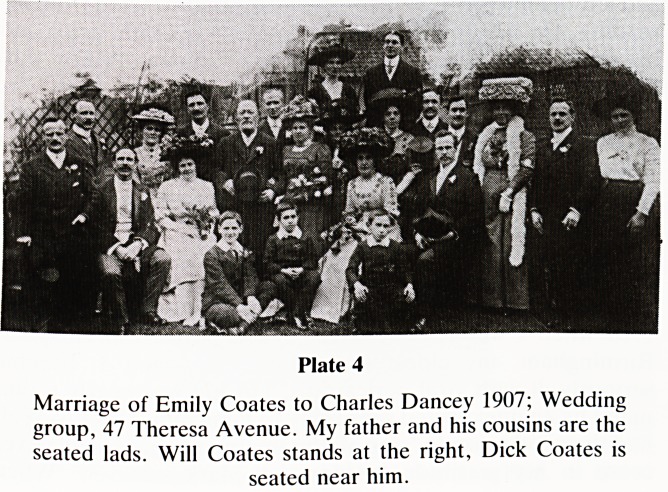


**Plate 5 f5:**
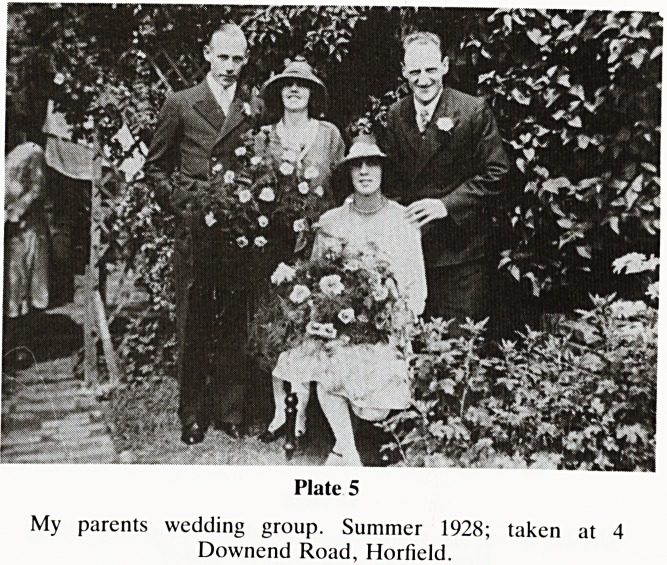


**Plate 6 f6:**
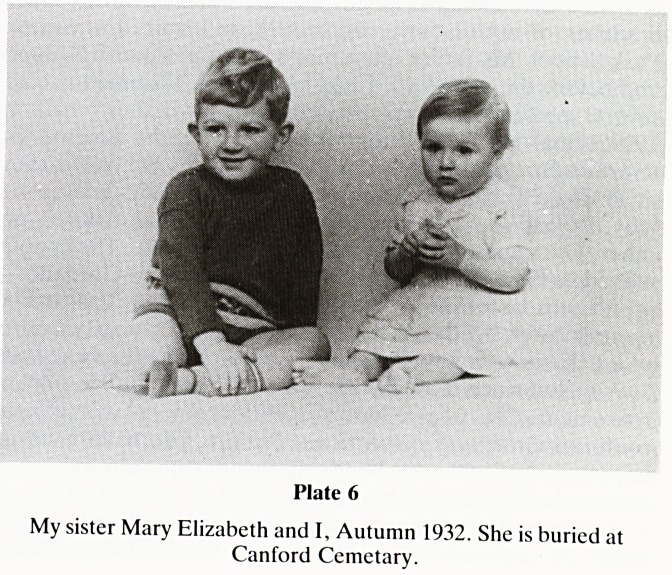


**Plate 7 f7:**
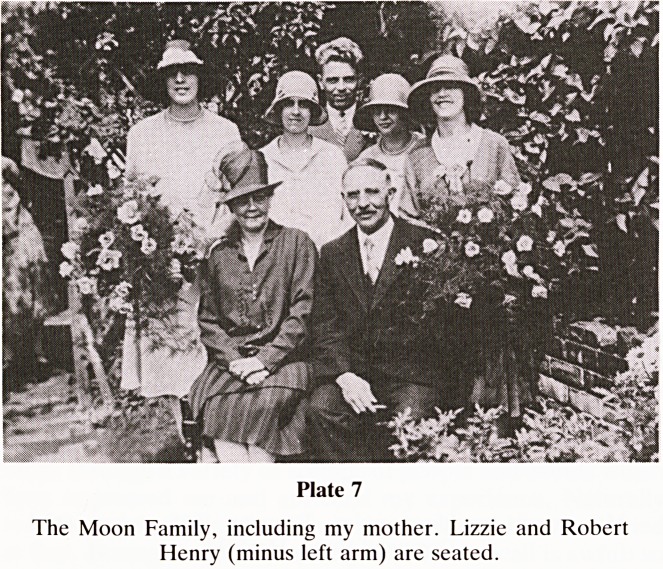


**Plate 8 f8:**
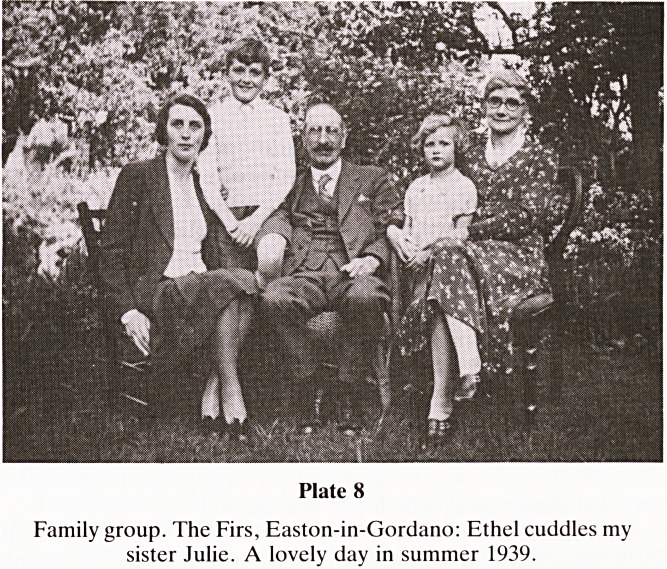


**Plate 9 f9:**
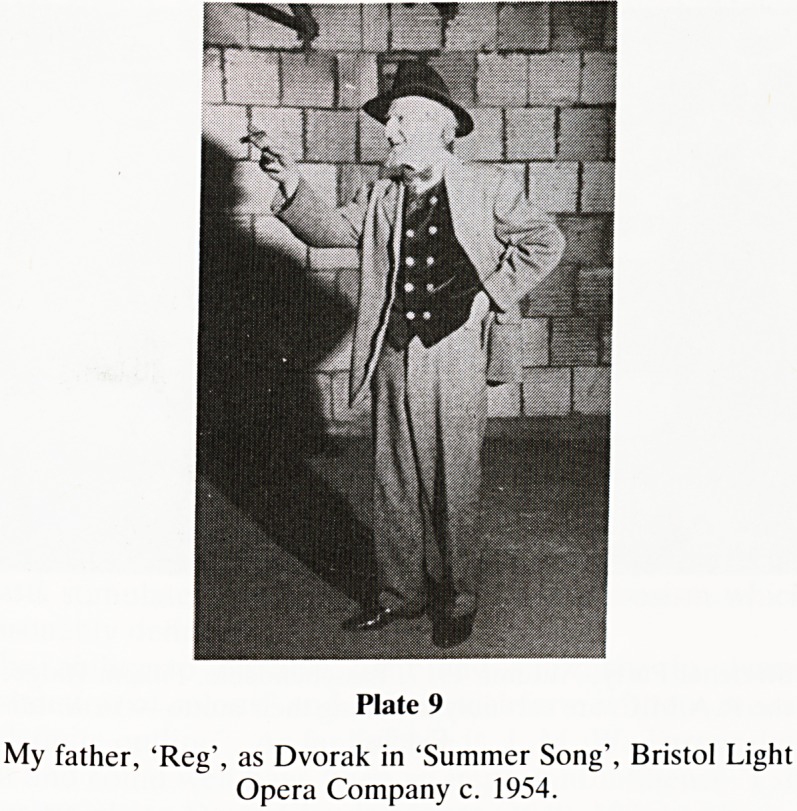


**Plate 10 f10:**
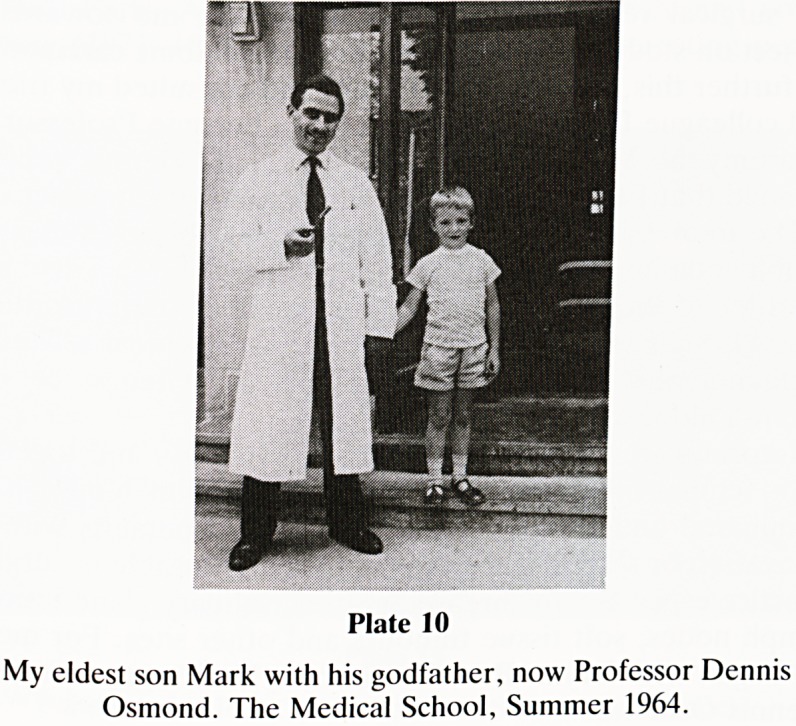


**Plate 11 f11:**
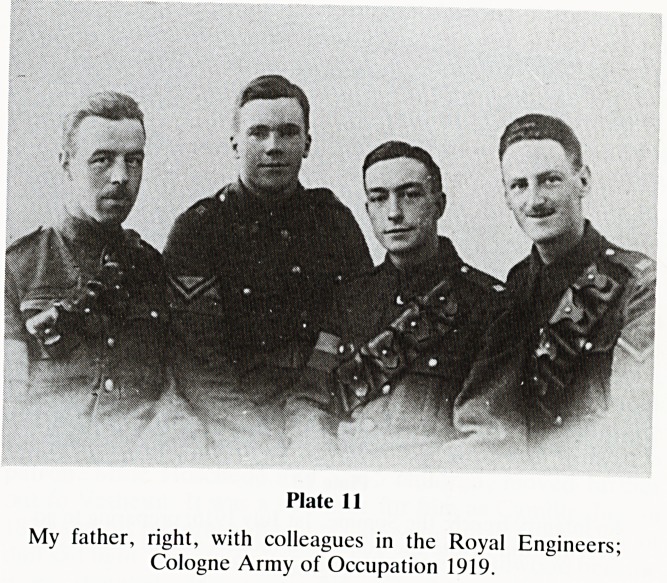


**Plate 12 f12:**
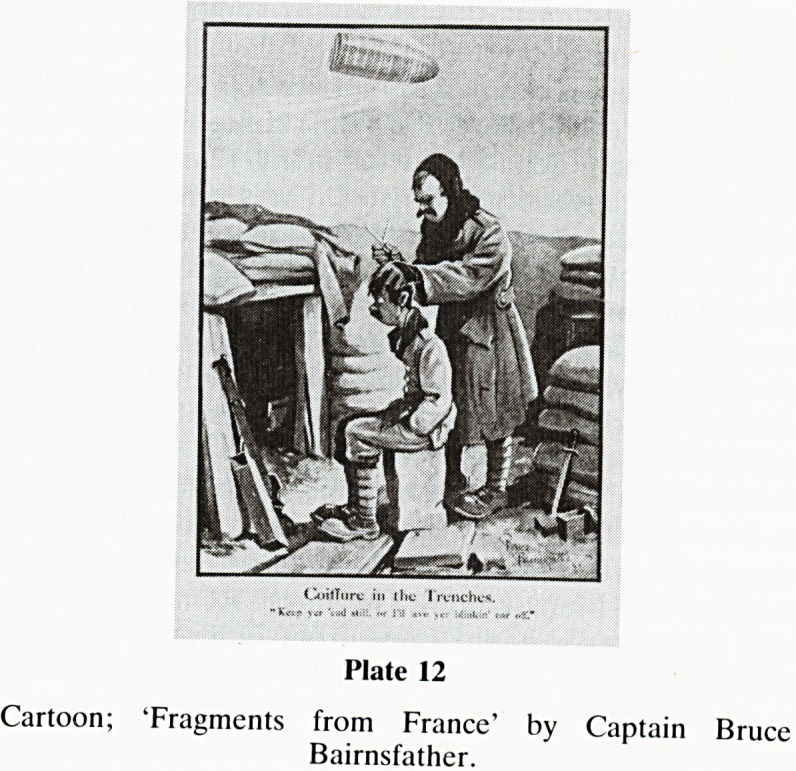


**Plate 13 f13:**
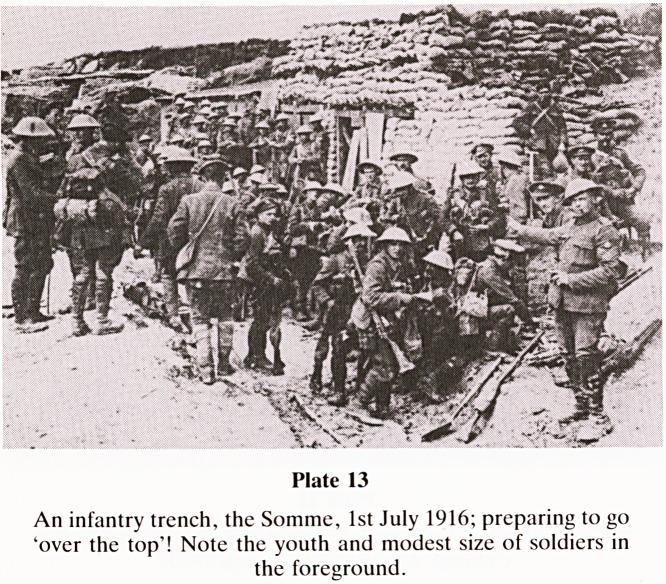


**Plate 14 f14:**
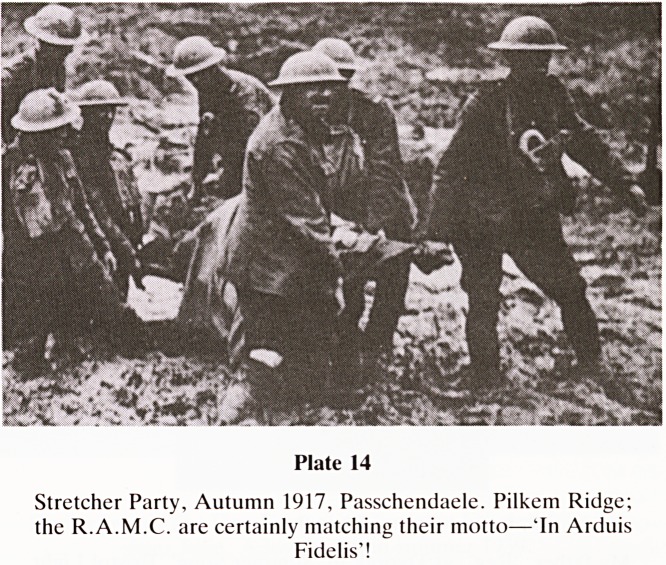


**Plate 15 f15:**
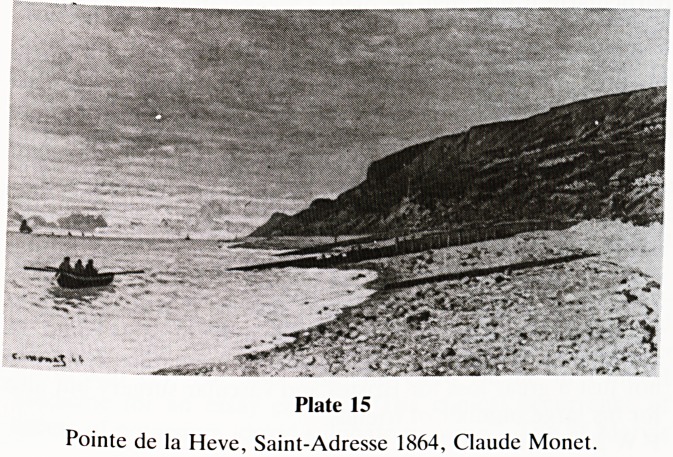


**Plate 16 f16:**
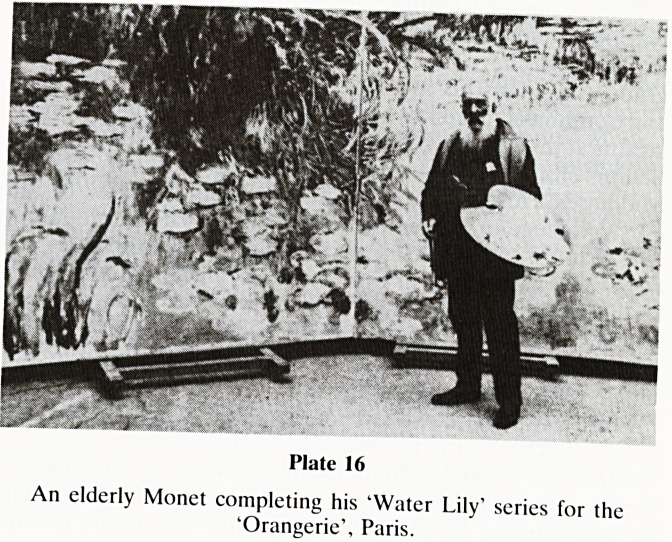


**Plate 17 f17:**
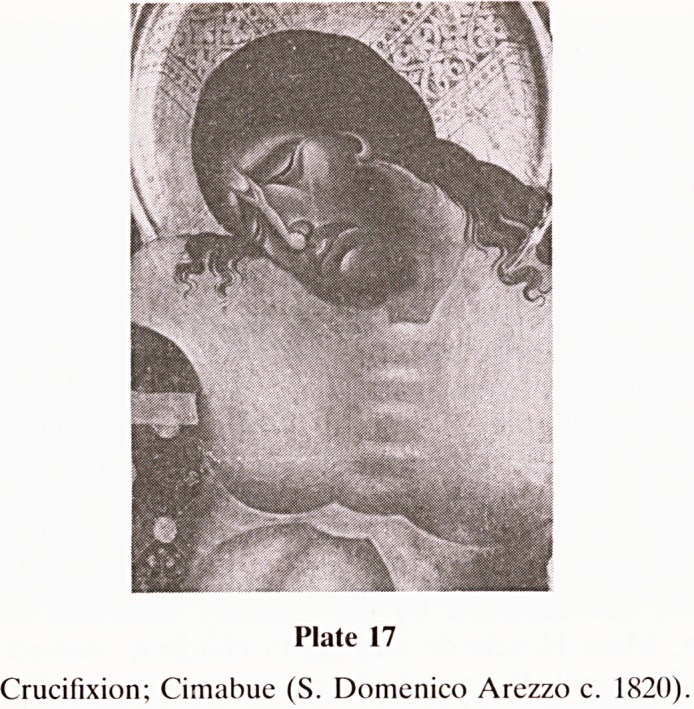


**Plate 18 f18:**